# Antimetastatic and antitumor activities of oncolytic NDV AMHA1 in a 3D culture model of breast cancer

**DOI:** 10.3389/fmolb.2024.1331369

**Published:** 2024-08-30

**Authors:** Ahmed Majeed Al-Shammari, Marwa Ibrahim Salman

**Affiliations:** ^1^ Experimental Therapy Department, Iraqi Center for Cancer and Medical Genetic Research, Mustansiriyah University, Baghdad, Iraq; ^2^ Department of Biotechnology, College of Science, University of Baghdad, Baghdad, Iraq

**Keywords:** 3D spheroids, Newcastle disease virus, scaffold, PKH67 linker, oncolytic virotherapy

## Abstract

**Introduction:**

Newcastle disease virus (NDV) AMHA1 is capable of killing cancer cells by direct replication or induction of apoptosis alongside other pathways. In this study, we report the potent antimetastatic and anticancer activities of NDV AMHA1 in a 3D spheroid model of breast cancer metastasis.

**Methods:**

we used two breast cancer cell lines AMJ13 and MCF7 in our metastasis model system.

**Results:**

First, we showed that NDV AMHA1 can infect and kill breast cancer cells in proliferating adherent cells and tumor spheroids using different virus doses and studying virus replication kinetics. We showed that NDV can infect and spread within the spheroids that represent metastasis before and after reattachment. Furthermore, we evaluated the ability of NDV to induce apoptosis in cancer spheroids and by virus tracking showed that NDV infection is essential for the elimination of these metastasis spheroids.

**Discussion:**

The mechanism by which NDV induces cell killing in the metastasis model is the induction of caspase-3 and P21 and inhibition of Ki67 in cancer cells, but not in normal cells. In conclusion, these results indicate that NDV AMHA1 has the ability to kill breast cancer metastases in suspension or attached, and this is a novel finding of NDV AMHA1 being a possibly efficient therapy against human metastatic breast cancer.

## Introduction

Breast cancer (BC) is a fatal cancer that affects women. Recently, it has surpassed lung cancer as the highest diagnosed cancer in women globally, with approximately 2.3 million new cases (11.7%), followed by lung (11.4%) and other types of cancers (Sung et al., 2021). In 2022, there were approximately 4.1 million women in the United States with a history of breast cancer. Approximately 4% of these women have metastatic breast cancer (Giaquinto et al., 2022). Treatments are crucial for eradicating and suppressing the metastases (Steeg, 2016). Most conventional therapies such as chemotherapies fail to eliminate breast cancer, and therefore, there is a great need for smart targeting therapy for breast cancer, and oncolytic virotherapy is a novel treatment approach for breast cancer (Javanbakht et al., 2023). Oncolytic virotherapy utilizes the selective ability of viruses to kill cancer cells and not harm normal cells, and it is developing toward being among conventional cancer therapies (Al-Shammari and Piccaluga, 2023). Oncolytic virotherapy is suggested to be effective against metastatic breast cancer. It can be developed to overcome common hurdles in treating metastatic breast cancer by enhancing systemic delivery, promoting intratumoral dissemination, and reducing the antiviral immune response. All these goals will improve the targeting of metastatic breast cancer cells (Cody and Hurst, 2015). Many oncolytic viruses were used for experimentally targeting breast cancer metastasis; for example, systemic administration of oncolytic replication-competent herpes simplex virus G47Δ in a mouse model by tail vein injection was shown to inhibit breast cancer cell growth, as well as its lung metastases (Wang et al., 2012). In a study using another version of the herpes simplex virus that encodes IL-12, it was found that in a local and systemic antibreast cancer *in vivo* model, it prevented lung metastasis (Ghouse et al., 2020). In addition, the efficacy of recombinant replicating and nonreplicating adenoviruses was also tested using an aggressive lung metastatic model of breast cancer, which needed more development to increase the antimetastatic effect (Ranki et al., 2007). Furthermore, oncolytic adenovirus targeting Wnt signaling inhibits the metastasis of liver cancer stem-like cells in an animal model (Zhang et al., 2017). Survivin-responsive conditionally replicating oncolytic adenovirus (CRAd) has antimetastatic properties *in vitro* in a chemotherapy-resistant metastatic human breast carcinoma model (Sakhawat et al., 2019). Xu et al. (2020) developed novel oncolytic adenoviruses with LyP-1-modification that target transforming growth factor β, which was tested in breast cancer mouse models and showed inhibition of tumor growth and metastases. Several other viruses were found to interfere with breast cancer metastasis in animal models and *in vitro*, such as coxsackievirus A21 (Skelding et al., 2009), the vesicular stomatitis virus (Ebert et al., 2005), and reovirus (Gebremeskel et al., 2021). Newcastle disease virus (NDV) AMHA1 induces oncolysis in breast cancer cells in a 3D coculture system, where it replicates in breast cancer cells while sparing normal cells, showing a high pattern of safety by being selective (Salman et al., 2022; Al-Shammari and Emran, 2022). NDV AMHA1 kills breast cancer cells through multiple, not classical mechanisms of action; first, it inhibits the glycolysis pathway by downregulating hexokinase and glyceraldehyde-3-phosphate, which leads to induction of apoptosis (Al-Shammari et al., 2019; Al-Ziaydi et al., 2020a; Al-Shammari et al., 2020). It has been shown to induce the caspase-dependent pathway through the expression of caspase-8 and 9 and the caspase-independent pathway by increasing the level of apoptosis-inducing factor and endonuclease G (Mohammed et al., 2019). Angiogenesis inhibition as a result of the virus taking over the cancer cell protein machinery was also observed (Al-Shammari et al., 2020). Newcastle disease virus works well in combination with conventional therapies such as papaverine and acarbose (Obaid et al., 2022; Akram et al., 2023); nanoparticles, such as gold (Jabir et al., 2023); as well as plant extracts such as alkaloid extracts of *Cyperus rotundus* L. (Al-Shammari et al., 2021). Breast cancer cells may gain direct access to systemic circulation through vein invasion. Polyclonal multicellular tumor cell aggregates spread significantly more efficiently than solitary cancer cells, possibly due to the physical trapping of large clusters in the circulation (Nathanson et al., 2022). During breast cancer metastasis, the macrometastases will no longer be dormant and will start to grow and evade the immune system. The macrometastases are tumors that are larger than 2 mm in diameter. The resumption of growth at a secondary location occurs due to the interactions between tumor cells and the microenvironment, which create a pre-metastatic niche (Szmulewitz et al., 2017).

To prevent breast cancer relapse through metastasis, we aim to target dormant tumor cells by oncolytic virotherapy. Using an *in vitro* 3D culture spheroid as the metastatic model, this study intends to evaluate the efficiency of NDV AMHA1 in eradicating these dormant tumor cells within the spheroids of breast cancer metastases.

## Materials and methods

### NDV proliferation and propagation

An attenuated NDV was grown in a chicken egg embryo (Al-Kindi, Baghdad, Iraq), viruses were isolated from the allantoic fluid of chicken eggs, and purification procedures were performed by centrifuging the fluid to remove debris. Centrifugation was performed at 3,000 rpm for 30 min at 4°C. After assessing hemagglutination (HA) as a quantitative technique, the NDV stock was aliquoted and stored at −80°C. Viral titers were determined in Vero–SLAM cells using a 50% infective dose (TCID 50) according to the standard procedure (Salih et al., 2017). The experimental procedures were carried out at the Experimental Therapy Department at the Iraqi Center for Cancer and Medical Genetics Research (ICCMGR), Mustansiriyah University, Baghdad, Iraq. The specific strain used in the study was the Iraqi AMHA1 strain of NDV.

### Cell lines and cell culture

The human breast cancer cell line AMJ13 (estrogen- and progesterone receptor-negative) was provided by the cell bank unit (Al-Shammari et al., 2015). The MCF-7 human breast cancer cell line (estrogen- and progesterone receptor-positive), Vero–SLAM, and monkey kidney cell line were provided by Dr. S. J. Russell Laboratory, Molecular Medicine Department, Mayo Clinic, United States. AMJ13 and Vero–SLAM were cultured in RPMI-1640 Medium. and MCF-7 cells and normal human-derived adipose tissue mesenchymal stem cells (hATMSCs) were grown in minimum essential medium (MEM) (US Biological, United States) with the addition of 10% (v/v) fetal bovine serum (FBS) (Capricorn Scientific, Germany) and 1% (v/v) penicillin–streptomycin (Capricorn Scientific, Germany). The cells were then placed in a humidified environment with 5% CO_2_ at a temperature of 37°C.

### Virus infection of cancer cells

**FIGURE 1:**
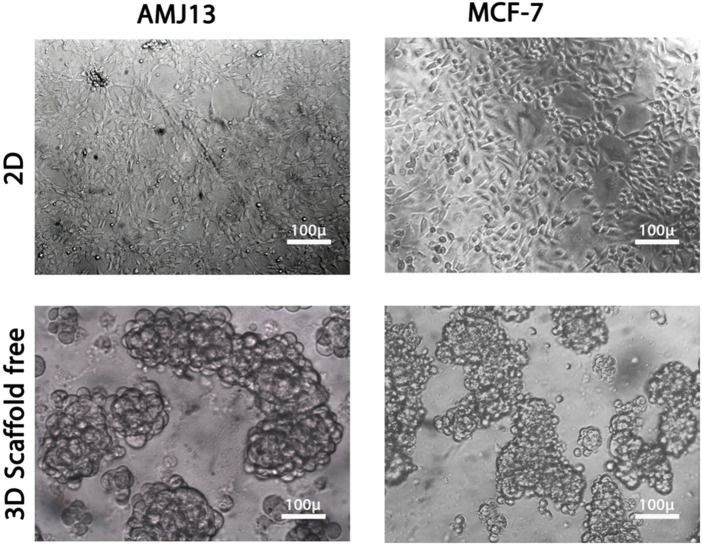
Comparison between two-dimensional (2D) and three-dimensional (3D) cell cultures. Both breast cancer cell lines, AMJ13 and MCF-7, grown in 2D and 3D culture. AMJ13 tends to form larger spheroids than MCF-7.

The first experiment was done to compare the virus killing efficiency against breast cancer cells in a 2D system with a 3D model of spheroids that represent tumor metastasis (Figure 1):

A. **2D adherent culture:** AMJ13 cells, MCF-7 cells, and hATMSCs were seeded in 5,000 cells/well in a 96-well plate and infected with NDV in multiplicity of infection (MOI) 1, 3, 5, 10, and 20. No virus (controls) was seeded in 1,000, 3,000, 4,000, and 5,000 cells/well for each type of cells. After 72 h of infection, the viability of the infected cells was evaluated using a WST viability assay (Elabscience Co., China) to determine the indirect quantification of dead cells (Akram et al., 2023).
B. **Generating 3D cancer cell spheroids:** AMJ13 cells, MCF-7 cells, and hATMSCs were seeded at 50,000 cells/well using controls as 10,000, 30,000, 40,000, and 50,000 cells on a 24-well cell floater plate (SPL3D ™ polystyrene microplates, Korea) and allowed to form spheroids for 3 days. Cell viability was assessed using a WST assay (Elabscience Co., China) to determine the indirect quantification of dead cells (M. I. Salman, Emran, and Al-Shammari, 2021).

### Kinetics of infection and cell viability

AMJ13 and MCF-7 cells were seeded using the same method as previously explained, both for spheroid and adherent cultures. The cells were then exposed to NDV with an MOI of 10, confirming optimal virus infection and synchronization of the virus life cycle in all cells. The viability of cultured cells at various time points, 24, 48, 72, 96, and 120 h, after infection for adherent and spheroid cultures was then determined using a crystal violet assay. To test the cell viability in the spheroids, after collection, the spheroids were pelleted, followed by crystal violet staining and trituration with a 26 gage needle (Feoktistova et al., 2016).

### Activation of NDV oncolysis after spheroid reattachment

The following procedure was adapted as described by Tong et al. (2015).

#### Infection of reattached spheroids

For reattached spheroids, AMJ13 and MCF-7 cell spheroids were formed by seeding 50,000 cells/well in a 24-well cell floater plate. These spheroids were moved to 6-well plates for the reattachment process. After 48 h of reattachment, the spheroids were infected at an MOI of 10. Spheroid images were taken 24 h after infection and then fixed and stained 72 h after infection using crystal violet stain.

#### Spheroid reattachment quantification

Cells were seeded at 50,000 cells/well on a 24-well cell floater plate to form spheroids over 72 h. Spheroids were infected with NDV at an MOI of 10. The spheroids were reattached by transferring to 6-well tissue culture plates. The spheroids were allowed to attach and disperse for an additional 72 h before fixing and staining with crystal violet stain. Dispersal areas were calculated using ImageJ 1.48 software (NIH) by subtracting the area of the core spheroid from the total area of the dispersion zone.

### Apoptosis study and morphological alteration

#### Evaluation of apoptosis (propidium iodide/acridine orange assay) in 2D culture

Apoptotic concentrations in spheroids (3D) and adherent (2D) cells (infected and controlled) in AMJ13 and MCF-7 breast cancer cells were measured by double staining with propidium iodide/acridine orange (PI/AO). Cells were seeded at a density of 7,000 cells/well in a 96-well plate the night before surgery and then treated with an NDV MOI of 10 for 72 h before staining with PI/AO and incubated at 37°C. For traditional PI/AO staining (10 μL AO + 10 μL PI + 1 mL of phosphate-buffered saline [PBS]), the tested wells received exactly 50 μL of the AO/PI stain mixture (at room temperature) for 30 s. Then, the dye was discarded. The photographs were taken using a Leica fluorescent microscope (Al-Shammari et al., 2019). In 3D spheroids, cells were prepared as mentioned above and then treated with NDV at an MOI of 10 for 72 h, in which the spheres were harvested and stained.

#### Evaluation of apoptosis in the 3D spheroid model

We assembled three to six spheroids and then incubated them at 37°C, 5% CO_2_, and 95% humidity. A 4 µM PI solution was prepared. A measure of 100 μL of the medium was removed from each well in the plate, making sure that the spheroids remained in place. To remove the medium, 100 µL of heated 1× PBS was added to each well, and then 100 µL of the liquid was removed (this washing step was repeated three times). Each well contained 100 µL of the PI solution. The plate was then covered with aluminum foil and incubated for 15 min at 37°C, 5% CO_2_, and 95% humidity. To reduce the background signal during the imaging, we repeated the first three washing methods. To image the spheroids, we used a fluorescence microscope (Rolver et al., 2019).

### Immunofluorescence assay

The cells were then exposed to standard washing steps (PBS three times), fixation (4% paraformaldehyde [PFA], 30 min, at RT), permeabilization (0.5% Triton-X, 30 min, at RT), and blocking (10% normal goat serum, 60 min). Cells were then treated with 1 µgmL^−1^ of each of the primary markers, KI67 (mouse monoclonal antibody, dilution 1:200; Elabscience, China), caspase-3 (mouse monoclonal antibody, dilution 1:200; Elabscience, China), and P21 (mouse monoclonal antibody, dilution 1: 500, Santa Cruz, CA, United States), which were diluted in blocking buffer and incubated for 1:30 h or overnight at 4°C. Cells were then treated with 1 µgmL^−1^ of secondary antibody Alexa Fluor 488 conjugated goat anti-rabbit IgG or Alexa Fluor 568 conjugated goat anti-mouse IgG for 2 h at RT. Cells were washed three times using PBS and mounted in VECTASHIELD with DAPI. Finally, they were examined under a fluorescence microscope.

### NDV fluorescence labeling

A total of 1.5 × 10^8^ NDV particles in PBS were labeled using the PKH67 Fluorescent Cell Linker Kit (Sigma-Aldrich, United States). For that, 1 µL of the PKH67 dye (Sigma, St. Louis, MO) was dissolved in 2 mL of diluent C. Two volumes of diluted PKH67 were mixed with one volume of the NDV suspension with pipetting. After 30 s, the labeling reaction was stopped by adding three volumes of the full medium and pipetting the suspension 5–6 times. The labeled virus was used for exposure (Balogh et al., 2011).

### Mock-infected control preparation

The PKH67–diluent C mixture was incubated with 1× PBS as described for virus labeling (without adding NDV). The reaction was stopped by adding a full medium, and then the mixture was used to cure the cells. Another control was presented using ordinary NDV without the PKH67 Fluorescent Cell Linker. In addition, another group has only spheroid cells.

### Statistical analysis

All our study results are presented as SD and mean ± SEM. The unpaired *t*-test and statistical analysis were performed using statistical software Excel version 10, GraphPad Prism version 7 (United States). The level of significance was established at *p* < 0.05.

## Results

### Virus infection of cancer cells

**FIGURE 2:**
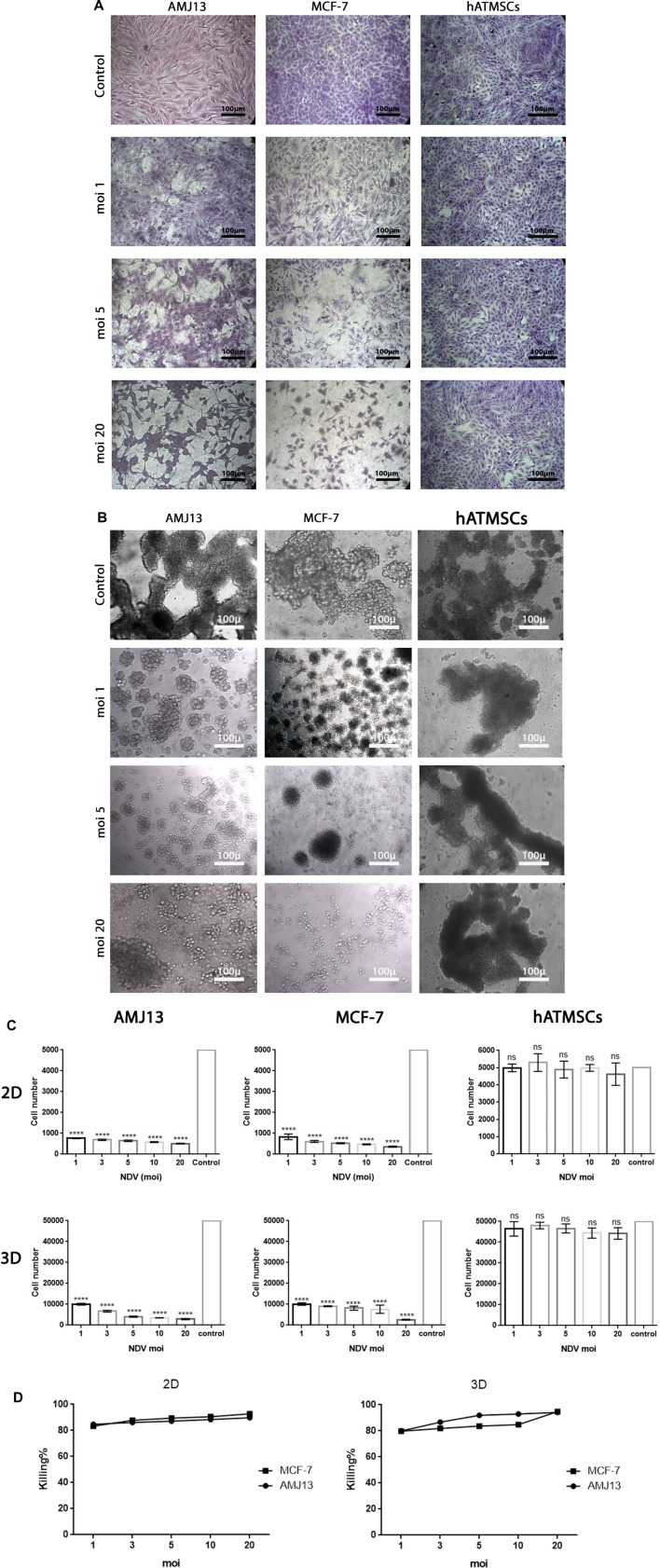
**(A)** Representative images of AMJ13 and MCF-7 2D culture under inverted microscopes for control non-infected cells compared to infected cancer cells showing the oncolytic effect of different MOIs causing a reduction in the number of cancer cells and a cytopathic effect. **(B)** Representative images of AMJ13, MCF-7, and hATMSC spheroids under inverted microscopes for control non-infected spheroids compared to infected spheroids showing the oncolytic effect of different MOIs causing a reduction only in cancer spheroid number and size, while the normal cell spheroids were not affected or have a negligible effect. **(C)** Analysis of NDV oncolytic-mediated killing of AMJ13 and MCF-7 cell lines in adherent and spheroid culture, while sparing normal hATMSCs. Viral infection of cancer and normal cells in the adherent (2D) culture system at 5,000 cells/well and in the spheroid (3D) culture system at 50,000 cells/well. Cells were seeded in ultra-low attachment dishes to form spheroids over 3 days. AMJ13 cells, MCF-7 cells, and hATMSCs were infected at increasing concentrations to a maximum of MOIs 1, 3, 5, and 10, and cell viability was measured after 72 h using a WST assay. The results in both 2D and 3D systems for cancer showed that NDV induced significant killing at all MOIs used, with increasing killing percentage with increasing virus dose, where we can see that the highest dose (MOI of 20) induced the highest killing percentage. At the same time, there are no or very low effects in normal mesenchymal cells. **(D)** Graphs showing both 2D and 3D models with exponential models reaching a plateau at high concentrations. This plateau can be considered the maximal efficacy point (MEP), representing the maximum response achievable with the drug for AMJ13 and MCF-7 cells.

We performed parallel viral infections of the adherent 2D cell culture system with established 3D spheroids in a suspension of breast cancer cells using different virus multiplicity of infections to compare the NDV oncolytic efficiency (Figure 1). In adherent 2D cultures, we found that NDV was capable of inducing oncolysis in both AMJ13 and MCF-7 cell lines with high killing capacities compared to the control in all MOIs tested (1, 3, 5, 10, and 20) (Figures 2A, C). On the other hand, the NDV oncolytic efficiency was evaluated in the 3D spheroid culture system in the same MOIs (1, 3, 5, 10, and 20) on both types of breast cancer cell line spheroids that represent a metastasis model. In both cell lines tested, the NDV exhibited a very potent oncolytic activity. In 3D spheroids, reductions in both the number and size of the spheroids were observed, which indicates that the NDV induces significant cell death, resembling the effect on the 2D culture system even at low MOI 1, in AMJ13 and MCF-7 spheroids compared to the control (Figures 2B, C). Interestingly, we observed a higher oncolytic efficiency of NDV in AMJ13 spheroids than in MCF-7. NDV infection resulted in significant cell death in both 2D and 3D culture models. The percentage of cells killed increased proportionally with the viral dose (MOI). The highest MOI tested (MOI of 20) induced the highest level of cell death. Investigating both 2D and 3D models with exponential models, we reach a plateau at high concentrations with MOIs of 10 and 20. This plateau can be considered the maximum efficiency point (MEP), representing the maximum response achievable with the drug for AMJ13 and MCF-7 cells (Figure 2D).

### Rapid kinetics of NDV-mediated killing of breast cancer cells

**FIGURE 3:**
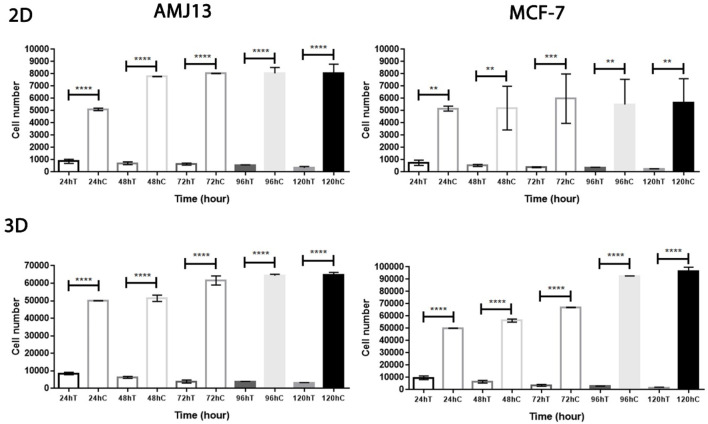
Rapid kinetics of NDV-mediated killing of AMJ13 and MCF-7 cell lines in the adherent and spheroid culture systems. NDV-mediated cell killing is observed within 24 h in 2D and 3D cell cultures but requires a longer incubation of cells to reach approximately complete NDV-mediated killing of cells. However, the oncolysis of adherent and spheroid culture reduced the viability due to NDV infection at 1–5 days.

The results of the first experiment show that AMHA1 NDV has potent oncolytic activity against both types of breast cancer cell lines (AMJ13 and MCF-7). This is assumed due to fast replication in cancer cells, completing several replication cycles in 72 h. We assessed this directly in 2D and 3D systems and compared viral infection kinetics for NDV in both breast cancer cell lines. Cells were infected with an MOI of 10 to ensure that all cells were completely infected from the beginning. We then analyzed cell viability to observe virus infection over 5 days. In 3D spheroids and 2D models, we found that NDV induced more than 80% oncolysis in both AMJ13 and MCF-7 cells within 24 h of infection (Figure 3), which was consistent with our previous findings (Figure 2C). Furthermore, more oncolysis of AMJ13 and MCF-7 spheroids continued until the end of 5 days. From this experiment, we conclude that NDV is effective in inducing oncolysis in spheroids and adhering culture as early as 24 h, and this effect is not reversed until the end of the 5 days after infection (Figure 3).

### Activation of NDV oncolysis after spheroid reattachment

The spheroidal reattachment assay was used to model metastasis formation due to the adhesion of spheroids to secondary sites. There were two models; the first one is the infection of the metastasis before the adhesion and the second is the infection of the metastasis after the adhesion in the secondary site.

### Infection-reattached spheroids

**FIGURE 4:**
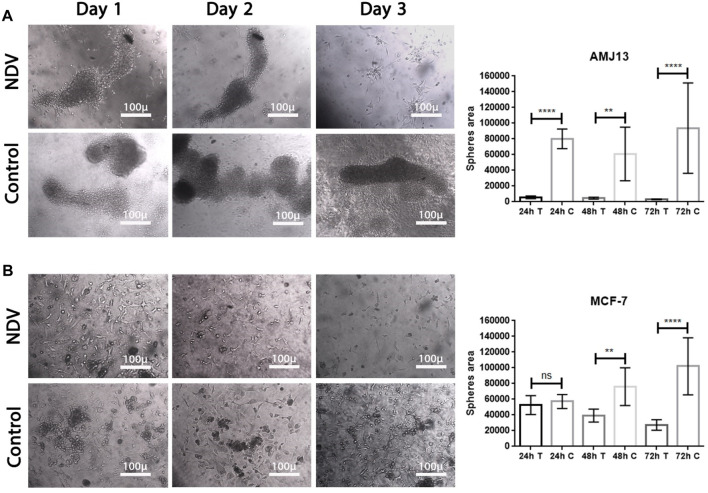
Reattached cancer spheroids. Cells were seeded to form spheroids and transferred to adherent culture to make them susceptible to NDV infection, **(A)** representing the infection of reattached AMJ13 spheroids at 24, 48, and 72 h, respectively, compared to control. **(B)** representing the infection of reattached MCF-7 spheroids at 24, 48, and 72 h, respectively, compared to control. The spheroids disperse 72 h after being infected with NDV. NDV infection is more effective in reducing viable cells of AMJ13 than MCF-7 spheroids. For all reattached spheroids, the mean dispersion area was quantified using ImageJ software. NDV exposure to each AMJ13 and MCF-7 reattaches spheroid cells.

**FIGURE 5:**
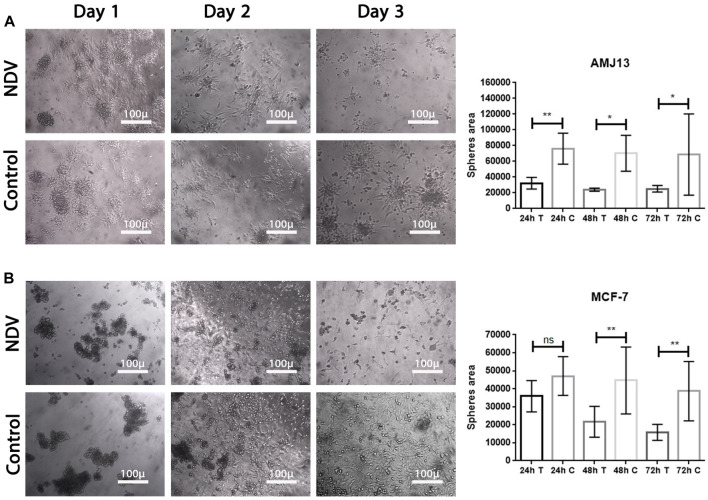
NDV oncolysis is reactivated after spheroid reattachment. **(A)** Representative images of AMJ13 spheroid infection, followed by reattachment to standard 2D tissue culture treated plastic. Spheroid reattachment was completely absent compared to no virus (control). **(B)** Representative images of the quantification of the dispersion area of MCF7 spheroids ×200. The mean dispersion area was quantified using ImageJ software (^*^ *p* < 0.05).

To facilitate the ability to evaluate the potential of NDV AMHA1 to target metastases, we examined the direct oncolytic effect on established reattached spheroids (Figure 4). The tumor spheroid area was measured by ImageJ after 72 h of treatment with NDV, and we allowed AMJ13 and MCF-7 cells to form spheroids and then infected them with NDV for 72 h. Infected spheroids were transferred to an adherent culture to allow reattachment for 72 h, then fixed, and stained; the area of the sphere was calculated. We observed NDV infection of the attached spheroids in both AMJ13 (Figure 5A) and MCF-7 cell lines (Figure 5B). As observed under an inverted microscope, there was a sound cytopathic effect in both cell lines, in the dispersing adherent cells emanating from the attached spheroids, with the spheroid itself being completely destroyed at the end of the third day in AMJ13 spheroids, while there was less effect on MCF7 spheroids. We could see that spheroids in the control are continuing to grow actively. These findings indicate that NDV induces oncolysis in metastases actively.

### Spheroid reattachment quantification

Breast cancer spheroids of both cell lines (AMJ13 and MCF-7) were infected before transfer to the adherent culture for spheroid reattachment (Figure 5). Spheroids were allowed to reattach and grow for another 72 h, after which the area of the spheres was measured. There was less killing of spheroids while in suspension; NDV AMHA1-mediated oncolysis was more aggressive once the spheroid was reattached and significantly reduced the ability of cells to grow out of the spheroids and form a viable monolayer on the third day of reattachment (Figures 5A, B).

### Newcastle disease virus induces apoptosis in spheroids and in adherent culture

**FIGURE 6:**
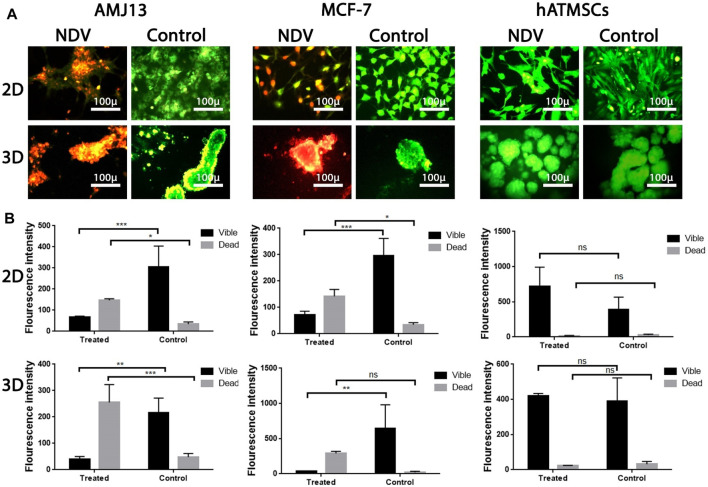
Investigation of NDV therapy to induce apoptosis in infected cells using acridine orange/propidium iodide assay: **(A)** AMJ13, MCF-7, and hATMSCs in adherent 2D and spheroid 3D cell culture indicated that NDV forced apoptosis, as proven by the presence of red-stained cells and untreated control cells emitting green fluorescence. AMJ13 and MCF-7 in both 2D and 3D culture cells showed that the number of apoptotic cells is higher, while there was no effect against the hATMSCs by NDV; ImageJ was used to calculate the intensity of the fluorescent dye in **(B)**. Values represent the (mean ± SEM). ^*^ *p* < 0.05, ^**^ *p* < 0.01, ^***^ *p* < 0.001, and ^****^ *p* < 0.0001. The magnification of all images was × 400.

The ability of NDV AMHA1 to induce apoptosis in 3D spheroids as a metastasis model and in 2D adherent cultures was evaluated and compared to that of normal human stem cells, as they have the ability to form spheroids *in vitro* to measure NDV safety in addition to its oncolytic activity against breast cancer cells. The AO/PI assay, as observed under the fluorescent microscope, demonstrated that untreated control cells appeared green (viable cells), while apoptotic cells treated with NDV appeared yellow or orange (dead cells) (Figure 6). The number of apoptotic dead cells was higher in NDV-treated spheroids and adherent cultures for both breast cancer cell lines, while no or very few apoptotic dead cells were observed in the control normal hATMSCs (Figure 6A) compared to the other cancer cell lines treated with the virus. These results indicated that NDV has oncolytic activity against cancer cells, while safe to normal cells (Figures 6A, B). The morphological changes exhibited by the treated cells in both 3D and 2D models after 72 h of infection were attributed to an intense oncolytic effect against both cancer cell lines, characterized by the shrinkage of the spheroids in 3D cultures and loss of cells in adherent 2D cultures for the treated cancer cells, while there were no such changes in normal groups (Figures 6A, B).

### NDV AMHA1 specifically enters and replicates in a 3D breast cancer metastasis model without harming normal tissue

**FIGURE 7:**
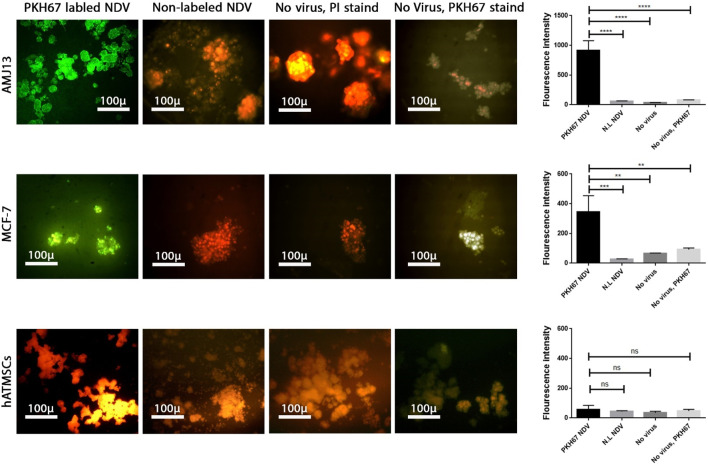
Labeling and tracking of virus particles. Spheroids were infected with the labeled NDV virus-carrying PKH67 linker. At 24 h after infection, cells were fixed and stained with propidium iodide (PI) for nuclear staining. PI staining is shown in red, and the cytoplasm is shown in green. Identification of NDV fusion sites by single-virus imaging carrying PKH67 linker (green) on AMJ13 and MCF-7 spheroids, respectively. Spheroid cells treated with non-labeled NDV in AMJ13 and MCF-7, respectively, and the nucleus of cells stained red from propidium iodide. There was spheroid cells left without any treatment as a control in AMJ13 and MCF-7. Finally, we had spheroid cells treated with the PHK67 cell linker as a second control in AMJ13 and MCF-7. All are imaged under a fluorescent microscope ×10.

To analyze NDV adsorption, penetration, and replication in the metastasis model of spheroids, PKH67-labeled virus particles (green fluorescent dye) were added to the spheroids with propidium iodide stain as the background stain. The entry of the virus into the infected cells was measured by measuring the fluorescence intensity and compared between the spheroids of cancer and normal cells (Figure 7). After 24 h, we observed a significant increase in green fluorescence intensity in breast cancer spheroids of both cell lines that were treated with labeled NDV compared to infected normal cells that did not show any significant green fluorescence and shows less virus entry and replication. Furthermore, spheroids exposed to PKH67 without NDV infection (labeling mixture containing no virus, mock PKH67) did not show significant green fluorescence, which confirms that labeled NDV is needed for the PHK67 dye to be inside the cells.

### Oncolytic NDV AMAH1 kills metastases through P21 induction and Ki67 inhibition

**FIGURE 8:**
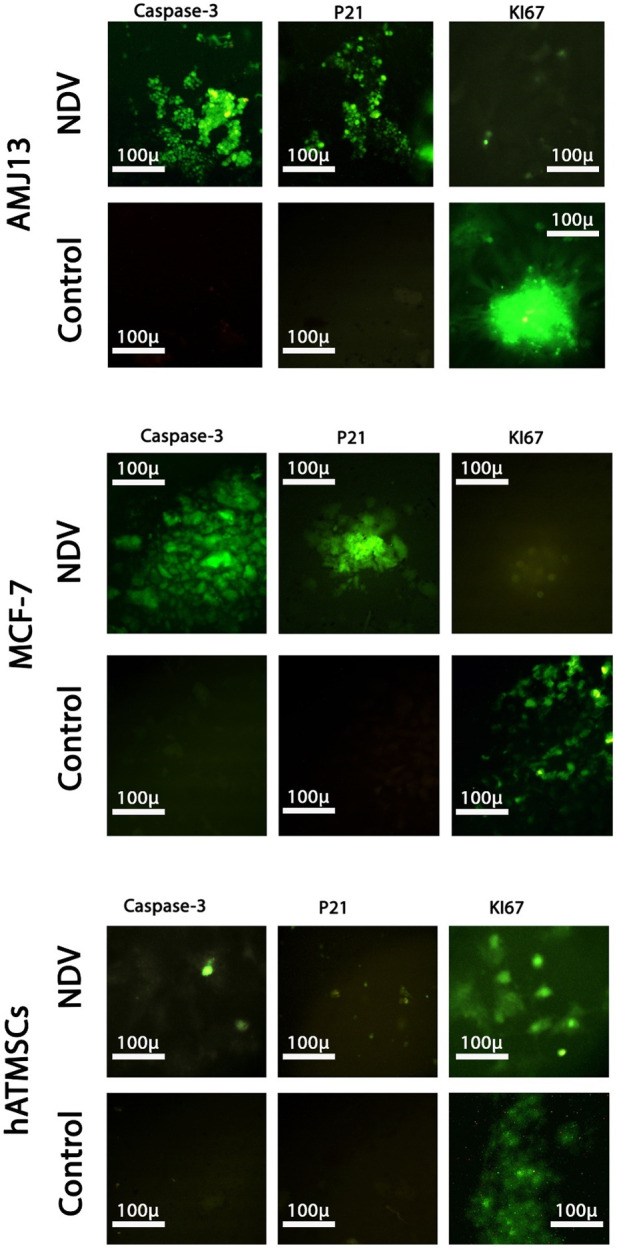
Analysis of the expressions of caspase-3, P21, and Ki67 by immunofluorescence assay in the 3D spheroids of the breast cancer metastasis model and normal tissue spheroids. Spheroids were infected with NDV (MOI 10) for 24 h; the spheroids were stained with anti-caspase-3, anti-P21, and anti-KI67 antibodies. Caspase-3 and P21 proteins exhibit a high expression of green fluorescence, while there is no expression in untreated spheroids (control). Furthermore, KI67 was absent in cancer spheroids treated with NDV. Normal tissue spheroids (hATMSCs) were not affected by the presence of virus as there were no notable changes in the expression of these proteins between treated and untreated spheroids, again confirming the safety of the virus.

To further investigate the molecular mechanism behind the killing effect induced by NDV AMHA1 in cancer cells and compare it to the effect of normal cells, we analyzed the expressions of caspase-3 (to confirm apoptosis), P21 (as tumor suppressor protein), and Ki67 (as proliferation marker). We found that there was a clear pattern of expression of these proteins in the cancer spheroids of both breast cancer cell lines, with significant differences in the expression level of these proteins compared to the untreated control spheroids. Moreover, a comparison to normal cells exhibited a clear opposite behavior for the NDV-infected spheroids (Figure 8). There was a high expression of the apoptotic protein caspase-3 and P21 in both AMJ13 and MCF-7 spheroids that were treated with NDV, unlike untreated spheroids. However, the expression of Ki67 proliferation markers was absent in NDV-treated cancer spheroids compared to untreated spheroids that showed high levels of expression. The expression of these proteins was not affected by the treatment with the virus, which explained that virus entry was low and no replication was found inside normal cells, which confirms the previous results shown in Figures 6, 7. The summarized results report the ability of NDV to inhibit Ki67 and activate the apoptosis pathway by triggering P21, which leads to apoptosis, such as high expression of caspase-3 in the spheroid model of metastasis.

## Discussion

In this work, we used a metastatic 3D spheroid model and a 2D adherent culture system for two types of breast cancer cell lines AMJ13 and MCF-7. The MCF-7 cell line represents estrogen–progesterone receptor-positive breast cancer, while AMJ13 is an estrogen–progesterone receptor-negative breast cancer cell with weak or absent expression of her-neu 2 expression (Al-Shammari et al., 2015). As triple-negative breast cancers have less therapeutic choices and show a higher risk of metastasis (Giaquinto et al., 2022), AMJ13 was chosen to form the spheroid as a metastatic breast cancer model to represent this fatal breast cancer. Therefore, it is very important to have an *in vitro* model for breast cancer metastasis for drug discovery and development, such as 3D spheroids (Fröhlich, 2023). Our objective was to assess the potential of using NDV as a first-line treatment for metastatic breast cancer.

Our study results reported substantial efficacy of NDV AMHA1 in 2D and 3D cultured cells that showed the impact of NDV on cell survival and growth inhibition that can be translated as an effective antitumor agent. In 3D spheroids, there were reductions in both the number and size of the spheroids, indicating that NDV induced significant cell death, which resembles the effect on the 2D culture system even with a lower MOI of 1 in AMJ13 and MCF-7 spheroids compared to the control. Interestingly, we observed a higher oncolytic effect of NDV in AMJ13 spheroids compared to MCF-7 spheroids. This can be seen as a promising anti-breast cancer effect by the NDV AMHA1 strain, which is consistent with previous results obtained by our team that found that NDV AMHA1 can replicate in breast cancer cells and kill them by inhibiting the glycolysis pathway by downregulating the hexokinase enzyme (Al-Ziaydi et al., 2020b). By studying NDV kinetics, we found that NDV induced more than 80% of oncolysis in the AMJ13 and MCF-7 spheroids and adherent culture as early as 24 h after infection, which was in support of the results of the first experiment that measured different virus MOIs and showed to be effective at all concentrations tested due to the high virus replication capacity and infection ability in cancer cells to spread from infected cells to other cancer cells (Chu et al., 2019). Furthermore, more oncolysis of AMJ13 and MCF-7 spheroids continued until the end of 5 days. It was reported that an oncolytic NDV strain, such as the Malaysian strain AF2240, can induce cell death and apoptosis as early as a few hours after infection in MCF-7 human breast carcinoma cells (Ghrici et al., 2013). However, the nonvirulent NDV LaSota strain was shown to require more time to induce killing in human breast cancer cell lines (Hassan et al., 2020). Furthermore, 3D spheroids are more similar to *in vivo* tumors, and our current results of the superior activity of NDV against breast cancer spheroids confirm what other researchers found that in human breast cancer xenografts in the mouse model, the intratumoral virotherapy of wild-type NDV strains inhibits subcutaneous breast cancer growth in SCID mice (Yurchenko et al., 2018). As antitumor activity was confirmed, we aimed to investigate the antimetastatic ability of NDV AMHA1. The spheroid reattachment assay was used to model metastasis formation due to the adhesion of the spheroids to secondary sites. There were two models: one is the infection of the metastasis before adhesion and the other is infection of the metastasis after adhesion at the secondary site. Tong et al. (2015) used spheroid reattachment as a representative model for the intraperitoneal spread of cancer cells, which resembles the process observed in patients. To facilitate the ability to evaluate the potential of NDV AMHA1 to target metastases, we examined the direct oncolytic effect on established reattached spheroids. As observed under an inverted microscope, in both cell lines, the spheroids were completely destroyed by the end of the experiment. This confirms that NDV induces oncolysis in metastasis actively. In the second model, the spheroids were infected before transfer to adherent culture for spheroid reattachment. Spheroids were allowed to reattach and grow for another 72 h, after which the area of the spheres was measured. There was less killing of spheroids while in suspension; NDV AMHA1-mediated oncolysis was more aggressive once the spheroid was reattached and significantly reduced the ability of cells to grow out of the spheroids. The current study provides a novel finding and is the first to confirm the antimetastatic effect of NDV in an *in vitro* model for breast cancer metastasis in hormone-dependent and independent breast cancer cells. Virotherapy using different viruses was tested as the breast cancer antimetastatic therapy, such as the measles virus, which inhibits human breast cancer metastases and improves the survival of the xenograft model (Jing et al., 2015). Furthermore, NDV AMHA1 induced apoptosis in 3D spheroids representing the metastasis model, as well as in 2D adherent cultures without induction in normal human stem cell spheroids, which confirms the safety of NDV in addition to its oncolytic activity against breast cancer cells. Apoptosis is a key cell death mechanism pathway that is caused by a suicide program in which cells on the verge of death release intrinsic enzymes that kill the nuclear DNA of the cells, as well as nuclear and cytoplasmic proteins along with active caspases that manage the proportions of various cell types (Bhattacharyya et al., 2014). The greatest inducer of apoptosis, according to the AO/PI test, was NDV treatment, which was consistent with our previous findings. In cells infected with NDV, apoptosis occurs (Arora et al., 2015). Apoptosis is an indispensable action to inhibit tumor development and cancer cell growth (García-Castillo et al., 2017). In both spheroids of the cell lines studied, the AO/PI apoptosis assay revealed that NDV is an efficient therapy for selectively inducing apoptotic cell death in cancer cells compared to normal cells. The Iraqi NDV strain causes DNA fragmentation of cancer cells and a higher level of caspase-3, leading to cell death (Al-Shammary et al., 2014; Al-Shammari, 2018). We found that NDV AMHA1 enters and replicates in breast cancer metastases specifically without harming normal spheroids. This was measured by labeling the NDV particles with the PKH67 dye. We observed a significant increase in the intensity of green fluorescence in infected breast cancer spheroids after 24 h, while they did not show any significant green fluorescence in infected normal cells, confirming fewer virus entries and fewer replications. The PKH67 linker offers good information on virus entry, which helps us understand virus trafficking (Ewers and Schelhaas, 2012). The exact mechanism of the antimetastatic effect of oncolytic NDV AMHA1 on the elimination of breast cancer metastases was found to be upregulation of the P21 protein and the downregulation of the Ki67 protein. These protein levels in normal cells remain stable during the experimental infection with NDV, which again confirms the safety of NDV. The ability of NDV to inhibit Ki67 and activate the apoptosis pathway of P21 was followed by high expression of caspase-3 in the metastasis spheroid model. Studies investigating the cellular functions of Ki67 have suggested that its presence is essential for cell proliferation (Kausch et al., 2004; Rahmanzadeh et al., 2007). In an experiment using complementary oligonucleotides to Ki67 mRNA, it was shown that DNA synthesis was inhibited, as reported by Schlüter et al. (1993). Furthermore, a similar study described that microinjection of antibodies against murine Ki67 caused a significant reduction in the proportion of dividing cells (Starborg et al., 1996). The levels of the tumor suppressor protein, P21, increase in G1 in a p53-dependent way in response to DNA damage; p16; and p15, a recently identified p16-related inhibitor. p27 Kip1, a P21-related protein, has been reported to be a negative regulator of G1 advancement (Nakagawa et al., 2018; Lamolinara et al., 2020). The expression of P21 is induced by wild-type p53, but not mutant p53. p21 (WAF1/CIP1), a cyclin-dependent kinase inhibitor that attenuates the cell cycle (Singh et al., 2017). The caspase-3 level is elevated by NDV infection. Caspase-3 is an essential executioner molecule during the apoptotic process. Caspase-3 mechanisms involve the cleavage of several important cellular proteins that lead to cell death by apoptosis (Pu et al., 2017; Yang et al., 2018).

## Conclusion

Based on these findings, we propose that the oncolytic NDV AMHA1 has anti-breast cancer activity with high selectivity with the ability to destroy macrometastasis spheroids of breast cancer through virus replication and induction of apoptosis in the metastasis, making NDV AMHA1 strong candidates for clinical systemic breast cancer therapy.

## Data Availability

The original contributions presented in the study are included in the article/Supplementary Material; further inquiries can be directed to the corresponding author.
